# The holding temperature of blood during a delay to processing can affect serum and plasma protein measurements

**DOI:** 10.1038/s41598-021-85052-5

**Published:** 2021-03-22

**Authors:** Milton Ashworth, Benjamin Small, Lucy Oldfield, Anthony Evans, William Greenhalf, Christopher Halloran, Eithne Costello

**Affiliations:** Department of Molecular and Clinical Cancer Medicine, University of Liverpool, Ashton Street, Liverpool, L69 3GE UK

**Keywords:** Biomarkers, Blood proteins

## Abstract

Accurate blood-borne biomarkers are sought for diagnosis, prognosis and treatment stratification. Consistent handling of blood is essential for meaningful data interpretation, however, delays during processing are occasionally unavoidable. We investigated the effects of immediately placing blood samples on ice versus room temperature for 1 h (reference protocol), and holding samples on ice versus room temperature during a 3 h delay to processing. Using Luminex multi-plex assays to assess cytokines (n = 29) and diabetes-associated proteins (n = 15) in healthy subjects, we observed that placing blood samples immediately on ice decreased the serum levels of several cytokines, including PAI-1, MIP1-β, IL-9, RANTES and IL-8. During a delay to processing, some analytes, e.g. leptin and insulin, showed little change in serum or plasma values. However, for approximately half of the analytes studied, a delay, regardless of the holding temperature, altered the measured levels compared to the reference protocol. Effects differed between serum and plasma and for some analytes the direction of change in level varied across individuals. The optimal holding temperature for samples during a delay was analyte-specific. In conclusion, deviations from protocol can lead to significant changes in blood analyte levels. Where possible, protocols for blood handling should be pre-determined in an analyte-specific manner.

## Introduction

Performing blood tests for diagnostic purposes is routine practice within modern healthcare and blood collected by phlebotomy is the most frequent human sample sent for analysis^1^. Consistent with this, novel accurate and cost-effective blood-borne biomarkers are avidly sought for a variety of clinical indications, including early disease detection, diagnosis, prognosis and treatment stratification. With a large quantity of both molecular and cellular information pertaining to the entire body, blood is an attractive source of biomarkers. It is minimally invasive to obtain, changes in response to altered physiological states, and clinical laboratories are set up to accommodate it for diagnostic purposes. However, variations in handling during blood collection or processing can cause alterations in protein and metabolite profiles^2–4^, potentially leading to misinterpretation. Consequently, standardisation of sample collection processing and storage are essential in order to minimise bias and to optimise opportunities to discover new biomarkers.

Once removed from the body, blood degrades rapidly^5^, meaning that timely processing is critical. Delays, however, are sometimes inevitable and opinion varies on whether blood should be cooled or maintained at room temperature during a delay. Holding whole-blood samples at 4 °C for either 1 h or 4 h prior to processing has been shown to cause fewer changes in plasma cytokine levels compared to holding samples at room temperature for the same time intervals^6^. Cooling blood however, can have unwanted consequences. Platelets, for example, rapidly aggregate at 4°C^7^ and lose membrane integrity^8^, potentially affecting analyte measurement. Importantly, platelet aggregation does not occur when whole blood is held at room temperature^9^. For clinical biomarker discovery studies or large clinical study collections, practical decision-making is required regarding how best to maintain blood sample integrity, all the while accommodating the demands of busy Good Clinical Practice (GCP)/The Clinical Laboratory Improvement Amendments (CLIA) laboratories. Here, we investigated the effects of delays to sample processing on the levels of blood proteins, using cytokines and diabetes-associated proteins as examples. Specifically, we investigated the effects of holding blood at 4 °C or at room temperature prior to processing blood to obtain either serum or plasma.

## Results

To investigate the consequences of delay-induced deviations from standard protocols prior to serum or plasma processing, freshly obtained blood was subjected to a reference protocol, namely leaving blood at room temperature for 1 h prior to processing by centrifugation, and up to three alternative protocols (Table 1).

**Table 1:** Handling protocols. Blood samples (serum and plasma) were subjected to Incubation 1 only (Reference and Ice protocols) or subjected to Incubation 1 followed immediately by Incubation 2 (RT 4 h and RT + Ice protocols) prior to processing and storage at −80 °C.

Protocol name	Incubation 1	Incubation 2 (holding/delay)
RT (Reference)	Room Temperature for 1 h	None
RT 4 h	Room Temperature for 1 h	3 h at Room Temperature
RT + Ice	Room Temperature for 1 h	3 h on Crushed Ice
Ice	Crushed Ice for 1 h	None

The reference protocol is well established for serum. Although plasma preparation does not require a pre-centrifugation incubation time, we applied the same 1 h at room temperature reference protocol, to allow for transit time to the laboratory and to accommodate busy laboratory environments where immediate centrifugation may not be possible. We considered two pre-processing holding/delay temperatures (Incubation 2, Table 1), room temperature (RT) and 4 °C (Ice), and investigated the effect of a 3 h delay in blood pre-processing. Concerned that standard operating procedures (SOP) steps which include the use of ice or refrigeration could lead to the inadvertent immediate placing of collection tubes at 4 °C, we also examined the consequences of immediate incubation of samples on ice (Ice) for 1 h as an additional alternative protocol (Table 1). The concentrations of C-peptide, ghrelin, GIP, GLP-1, insulin, leptin, PAI-1, resistin, visfatin, IL-8, IL-9, IFN-g, MIP1-β, PDGF-BB and RANTES were measured.

### Effects on serum analyte levels of cooling samples during processing

**Figure 1:**
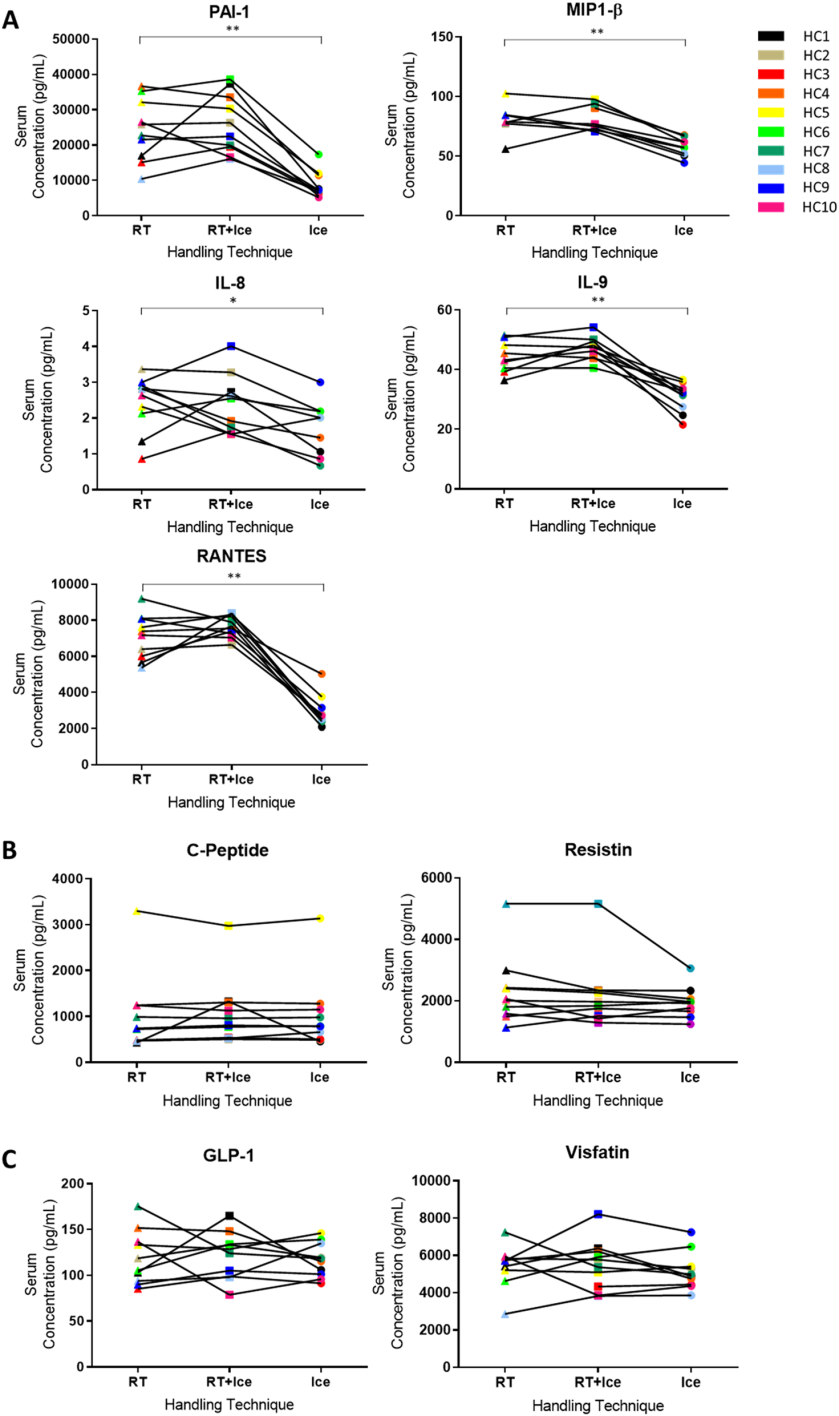
The effects of alternative pre-processing protocols on serum analyte levels. The serum levels of selected analytes in healthy control samples following three pre-processing protocols are shown. The reference protocol, 1 h at room temperature (RT) and two alternative protocols, 1 h at RT followed by a further 3 h on ice (RT + Ice) or 1 h on ice (Ice) were compared. (**A**) Significant differences were identified in the levels of PAI-1, MIP1-B, IL-9, RANTES and IL-8 levels. (**B**) Some analytes demonstrated minimal changes. (**C**) While no statistically significant differences are observed for some analytes, specific individual samples undergo considerable changes in response to handling variables. Paired t-tests were performed to examine for significant differences between mean analyte levels in samples from reference and alternative protocols. * represents *p* ≤ 0.05, ** represents *p* < 0.01.

The Ice protocol led to an increase in sample viscosity resulting in thick, gelatinous serum that was difficult to handle. For this reason, this protocol was not extended beyond 1 h. Moreover, this was accompanied by significantly lower serum levels of PAI-1, MIP1-β, IL-9, RANTES and IL-8 compared to the levels observed in the reference protocol (RT) (*p* < 0.05; Fig. 1A, Supplementary Table S1). There were fewer observed changes and no statistically significant differences in the mean serum analyte levels between the reference protocol and the RT + Ice protocol.

Some analytes, such as C-Peptide and resistin (Fig. 1B) showed fairly consistent levels across each handling protocol for most individuals. However, for other analytes, such as GLP-1 and visfatin (Fig. 1C), considerable variations in concentration were observed for the samples of several individuals in the study in response to different handling protocols. However, because the direction of change varied from individual to individual, paired t-tests of the mean levels per group showed no significant differences, masking substantial individual changes clearly observable in the paired analysis graphs (Fig. 1, Supplementary Figure S1). We therefore undertook an alternative data analysis approach. For each analyte, the median concentration measured in the reference protocol was used to classify individuals as either high (≥ median level) or low (< median level) for that analyte. Receiver-operating characteristic (ROC) curve analysis was then used to assess the accuracy of classification of individuals as high or low for that analyte following sample processing according to the alternative handling protocols. In this manner, we tested whether hypothetical classification properties of each marker were robust across processing conditions.

For IL-8, PAI-1 and RANTES, area under the curve (AUC) values were low when serum samples were subject to the Ice protocol (Table 2, Supplementary Figure S5A). This is consistent with our observation (Fig. 1A) that the Ice protocol markedly affected the levels of these analytes in serum. The measured concentrations for these analytes in the RT + Ice protocol produced AUCs of 0.7 and above (Table 2), confirming that this alternative protocol had less impact on individual variation than the Ice protocol. Consistent with the minimal changes observed in Fig. 1B, C-Peptide and resistin measurements resulted in AUCs of 0.7 and above, for both the RT + Ice and the Ice protocol. This was also true for ghrelin, GIP, insulin and leptin (Table 2, Supplementary Figure S5B), indicating that these analytes were relatively resilient to the changes in sample processing in this study. The AUC values for IL-9 were above 0.7 in both protocols, despite the mean concentration being significantly decreased in the Ice protocol (Table 2, Fig. 1A, Supplementary Figure S5B). This indicates that for IL-9, despite the Ice protocol showing accurate classification of individuals in ROC analysis, this protocol cannot be used interchangeably with the reference protocol due to the consistent decreases in IL-9 observed in individual samples. For analytes, such as GLP-1 and visfatin, where different handling protocols resulted in individual-specific alterations in analyte level (Fig. 1C, Supplementary Figure S5C), alternative protocols resulted in AUCs of less than 0.7, indicating that these protocols could lead to misclassification of individuals compared to their classification in the reference protocol. This was also the case for MIP-1b, PDGF-bb and IFN-γ (Table 2).

**Table 2:** Results of statistical and ROC classification analyses for each analyte, grouped by pre-processing condition.

Cytokine	Serum				Plasma			
	Ice		RT + Ice		Ice		RT + Ice	
	AUC	*p* value	AUC	*p* value	AUC	*p* value	AUC	*p* value
C-Peptide	0.960	0.940	0.840	0.940	0.950	0.583	0.950	0.583
Ghrelin	1.000	1.000	0.850	1.000	0.938	0.902	0.938	0.902
GIP	1.000	0.491	0.700	0.491	1.000	0.199	1.000	0.192
GLP-1	0.600	1.000	0.520	1.000	0.800	0.996	1.000	0.996
IFN-γ	0.000	0.981	0.042	0.981	0.960	1.000	0.460	1.000
IL-8	0.639	0.024	0.778	0.626	0.660	0.129	0.560	0.555
IL-9	0.800	0.000	0.700	0.077	0.500	0.099	0.400	0.020
Insulin	0.875	0.483	0.750	0.874	0.938	0.605	1.000	0.605
Leptin	1.000	0.279	0.875	0.512	1.000	1.000	1.000	1.000
MIP-1β	0.333	0.008	0.500	0.955	1.000	0.185	0.833	0.041
PAI-1	0.600	0.000	0.720	0.501	0.800	0.996	1.000	0.996
PDGF-bb	0.650	0.851	0.400	0.998	0.400	0.148	0.450	0.275
RANTES	0.550	0.000	0.700	0.351	0.280	0.146	0.720	0.000
Resistin	0.920	0.261	0.840	0.295	0.900	0.347	1.000	0.909
Visfatin	0.300	1.000	0.400	1.000	0.750	0.908	0.700	0.970

*p* values are for paired t-tests comparing the mean analyte levels in the reference protocol and the indicated protocol.

### Effects on plasma analyte levels of cooling samples during processing

**Figure 2:**
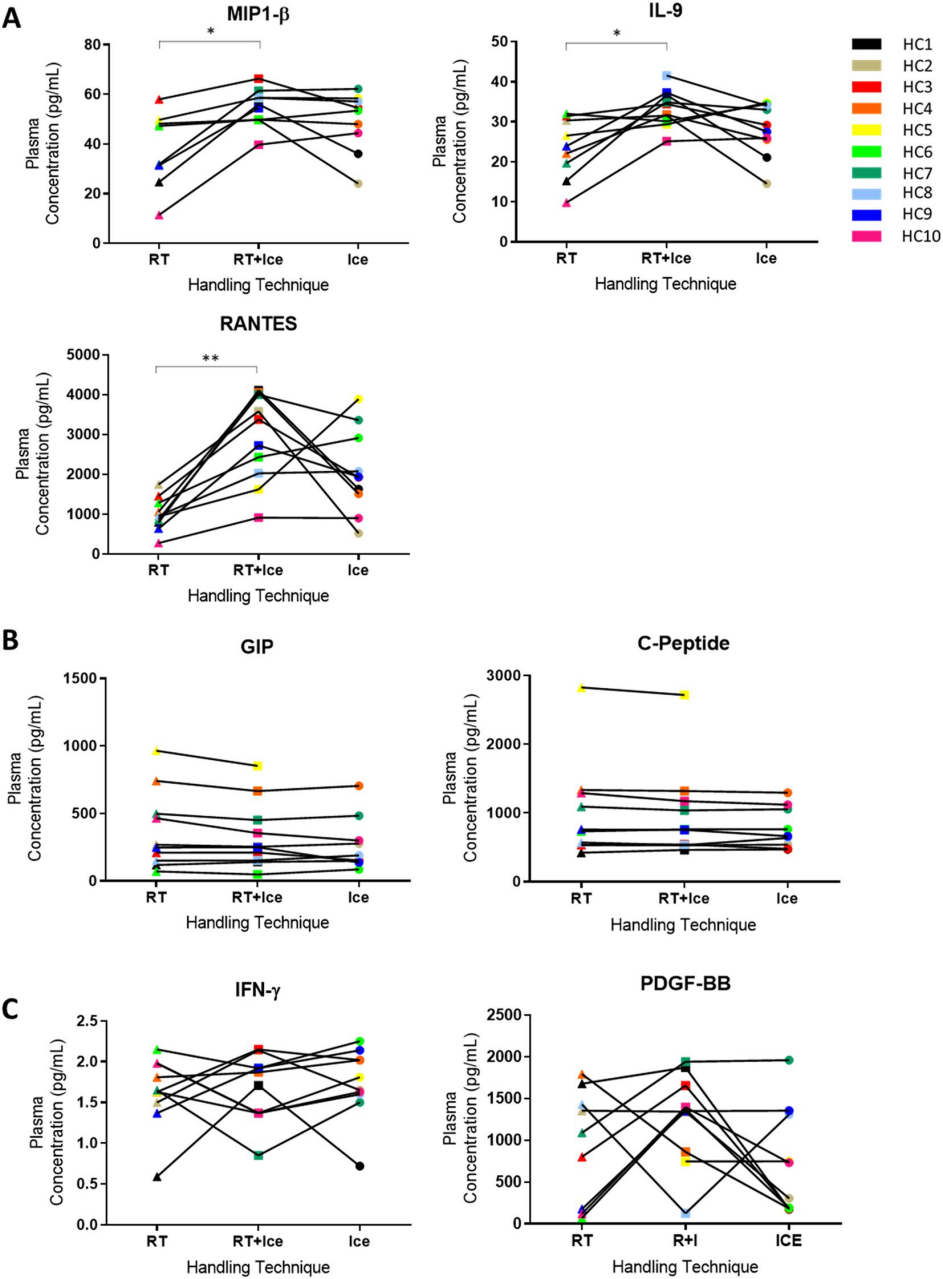
The effects of alternative pre-processing protocols on plasma analyte levels. The plasma levels of selected analytes in healthy control samples following three pre-processing protocols are shown. The reference protocol, 1 h at room temperature (RT) and two alternative protocols, 1 h at room temperature followed by a further 3 h on ice (RT + Ice) or 1 h on ice (Ice) were compared. (**A**) Significant differences were identified in the levels of MIP1-B, IL-9 and RANTES levels. (**B**) Some analytes demonstrated minimal changes. (**C**) While no statistically significant differences are observed for some analytes, specific individual samples undergo considerable changes in response to handling variables. Paired t-tests were performed to examine for significant differences between mean analyte levels in samples from reference and alternative protocols. * represents *p* ≤ 0.05, ** represents *p* < 0.01.

The Ice protocol did not result in significant changes in the mean concentrations of any analyte measured (Fig. 2, Supplementary Figure S2). However, the plasma protein concentrations of 3/16 analytes measured, MIP1-β, IL-9 and RANTES (Fig. 2A) were significantly increased if the sample was subjected to the RT + Ice protocol. Some analytes, such as GIP and C-Peptide (Fig. 2B) were largely unaffected by either of the alternative pre-processing protocols. The measured concentrations of other analytes, such as IFN-γ, and PDGF-bb (Fig. 2C) followed different patterns in distinct individuals, with the RT + Ice or Ice protocols causing increases in concentration for some individuals and decreases for others.

Despite these changes, the mean concentration of these analytes across the protocol groups remained unchanged prompting us to undertake ROC analysis as before. AUC values of 0.7 and above were achieved for RANTES during the RT + Ice protocol, suggesting this condition had less impact on individual variation than the Ice protocol (Table 2, Supplementary Figure S6A). In contrast, more individual variation was observed in the RT + Ice protocol for IFN-γ than the Ice condition (Table 2, Supplementary Figure S6B). AUC values (Table 2) of 0.7 and above were achieved following both the Ice and RT + Ice protocols for 10/15 analytes, namely C-Peptide, ghrelin, GIP, GLP-1, insulin, leptin, MIP-1β, PAI-1, resistin and visfatin (Table 2 Supplementary Figure S6C). This indicates that the changes in processing affected the accuracy of measurement of these analytes for a relatively small number of individuals only. For some analytes, such as GIP, C-Peptide, ghrelin, leptin, and resistin, protocol changes had minimal effects on the measured plasma values and hence on the accuracy of classification, with AUCs remaining exceptionally high (Table 2 Supplementary Figure S6C). By contrast, deviations from the reference protocol resulted in AUCs below 0.7 with considerable loss in the ability to classify individuals (Table 2, Supplementary Figure S6D) for IL-8, IL-9 and PDGF-bb. Our results suggested that placing plasma samples immediately on ice or holding them at 4° C in the event of a delay would have little or no impact on some analytes, but could change considerably the measured levels of others.

### Effects of holding serum samples at RT versus on ice during a pre-processing delay

**Figure 3:**
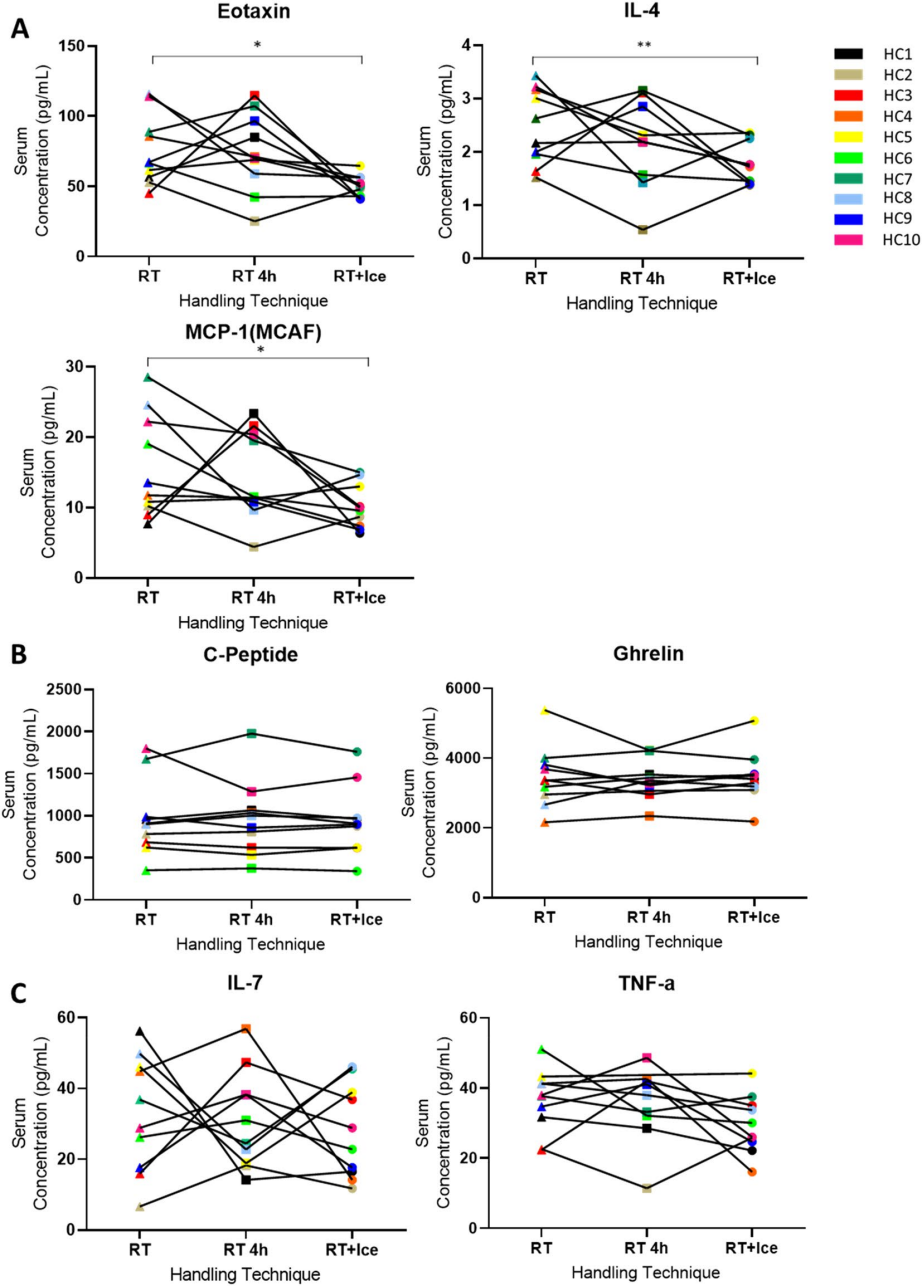
The effects of a delay and pre-processing protocols on serum analyte levels. The serum levels of selected analytes in healthy control samples following three pre-processing protocols are shown. The reference protocol 1 h at room temperature (RT) and two delay protocols, 1 h at room temperature followed by a further 3 h on ice (RT + Ice) or 1 h at RT followed by a further 3 h at RT (RT 4 h) were compared. (**A**) Significant differences were identified in eotaxin, IL-4 and MCP-1 levels. (**B**) Some analytes demonstrated minimal changes. (**C**) While no statistical difference are observed for some analytes, specific individual samples undergo considerable changes in response to handling variables. Paired t-tests were performed to examine for significant differences between mean analyte levels in samples from reference and alternative protocols. * represents *p* ≤ 0.05, ** represents *p* < 0.01.

We next investigated whether it was best to maintain samples at RT or on ice during a delay. We compared the outcome of processing without delay (reference protocol) with the outcome of processing samples held at RT for 1 h followed by an additional 3 h at RT (RT 4 h) versus RT for 1 h followed by 3 h on ice (RT + Ice) (Fig. 3, Supplementary Figure S3). Analytes were excluded if the coefficient of variance (CV) was above 20%, bringing the total number of analytes evaluated to 29. Compared to the reference protocol, significant reductions in the mean serum concentrations of eotaxin, MCP-1 and IL-4 were observed in the RT + Ice protocol, but not in the RT 4 h protocol (Fig. 3A, Supplementary Table S2). Some analytes appeared largely unchanged whether held at RT or 4 °C during a delay to processing (Fig. 3B), while for others the pattern of change was different in distinct individuals (Fig. 3C). ROC analysis indicated that for 15/29 analytes, alterations in individual responses to both of the delay protocols led to the misclassification of individuals resulting in AUC values of < 0.7 (Table 3, Supplementary Figure S7A). This included eotaxin, FGF-basic, glucagon, IL-12, IL-13, IL-17, IL-1β, IL-7, IL-9, IP-10, MCP-1, MIP-1α, MIP-1β, PDGF-bb, and RANTES. For six analytes, G-CSF, visfatin, ghrelin, IL-1ra, IL-2 and IL-4 the AUC values achieved in the RT + Ice protocol were ≥ 0.7 and were < 0.7 in the RT 4 h protocol, indicating that for these analytes, holding the sample on ice was preferable to holding it at room temperature during a delay (Table 3, Supplementary Figure S7B). However, the significant decrease in the concentration of IL-4 in the RT + Ice protocol (Fig. 3A), combined with the high AUC value, suggests ice caused a consistent decrease in this analyte in the individuals tested. This indicates that for IL-4, despite the good classification of individuals (high AUC) seen in the RT + Ice protocol, this protocol cannot be used interchangeably with the reference protocol. In contrast, for TNF-α, the AUCs were ≥ 0.7 in the RT 4 h protocol and < 0.7 in the RT + ICE protocol suggesting its concentration was more reliably measured when samples were held at RT, in the event of a delay to processing (Table 3, Supplementary Figure S7C). As before, some analytes, such as, leptin, insulin, resistin, and C-Peptide demonstrated little change in response to processing delays, regardless of holding temperatures (Table 3, Supplementary Figure S7D).

**Table 3:** Results of statistical and ROC classification analyses for each analyte, grouped by the delay condition.

Cytokine	Serum				Plasma			
	RT 4 h		RT + Ice		RT 4 h		RT + Ice	
	AUC	*p* value	AUC	*p* value	AUC	*p* value	AUC	*p* value
Eotaxin	0.640	0.907	0.600	0.014	0.280	1.000	0.280	1.000
C-Peptide	0.960	1.000	0.880	1.000	0.950	1.000	0.900	1.000
Visfatin	0.560	1.000	0.760	1.000	0.600	0.421	0.650	0.421
FGF-basic	0.583	0.794	0.667	0.794	0.550	0.850	0.275	0.850
G-CSF	0.333	0.293	0.917	0.090	0.650	0.296	0.550	0.347
Ghrelin	0.600	1.000	0.880	1.000	0.900	0.192	0.850	0.192
GIP	0.760	1.000	0.940	1.000	0.900	0.209	0.800	0.288
GLP-1	0.760	0.960	0.800	0.960	0.700	0.287	0.550	0.287
Glucagon	0.600	0.483	0.500	0.651	0.700	0.287	0.550	0.287
IL-12	0.475	0.937	0.100	0.735	0.580	0.313	0.640	0.876
IL-13	0.350	0.957	0.600	0.957	0.720	0.376	0.660	0.443
IL-17	0.500	0.983	0.650	0.183	0.520	1.000	0.500	1.000
IL-1β	0.306	0.792	0.444	0.792	0.560	0.246	0.720	0.817
IL-1ra	0.650	0.322	0.950	0.322	1.000	0.198	0.920	0.198
IL-2	0.475	0.975	0.975	0.748	0.950	1.000	0.850	1.000
IL-4	0.575	0.507	0.975	0.007	0.440	0.983	0.300	0.983
IL-7	0.320	0.807	0.680	0.798	0.720	1.000	0.420	1.000
IL-9	0.417	0.664	0.500	0.664	0.520	1.000	0.360	1.000
Insulin	0.920	0.367	0.960	0.367	1.000	0.883	1.000	0.883
IP-10	0.520	0.280	0.560	0.229	0.240	0.387	0.440	0.387
Leptin	1.000	0.617	1.000	0.920	1.000	0.186	1.000	0.186
MCP-1	0.440	0.661	0.680	0.024	0.640	1.000	0.480	1.000
MIP-1α	0.333	0.734	0.583	0.165	0.833	0.703	0.833	0.703
MIP-1β	0.563	0.669	0.563	0.532	0.450	1.000	0.300	1.000
PAI-1	0.800	0.987	0.760	0.542	0.650	0.989	0.850	0.989
PDGF-bb	0.438	0.594	0.563	0.200	0.280	0.948	0.520	0.948
RANTES	0.680	0.981	0.200	0.129	0.520	0.715	0.480	0.715
Resistin	1.000	0.259	0.960	0.259	1.000	1.000	0.950	1.000
TNF-α	0.750	0.927	0.450	0.166	0.500	1.000	0.833	1.000

*p* values are for paired t-tests comparing the mean analyte levels in the reference protocol and the indicated protocol.

### Effects on plasma analytes of holding samples at RT versus on ice during a pre-processing delay

**Figure 4:**
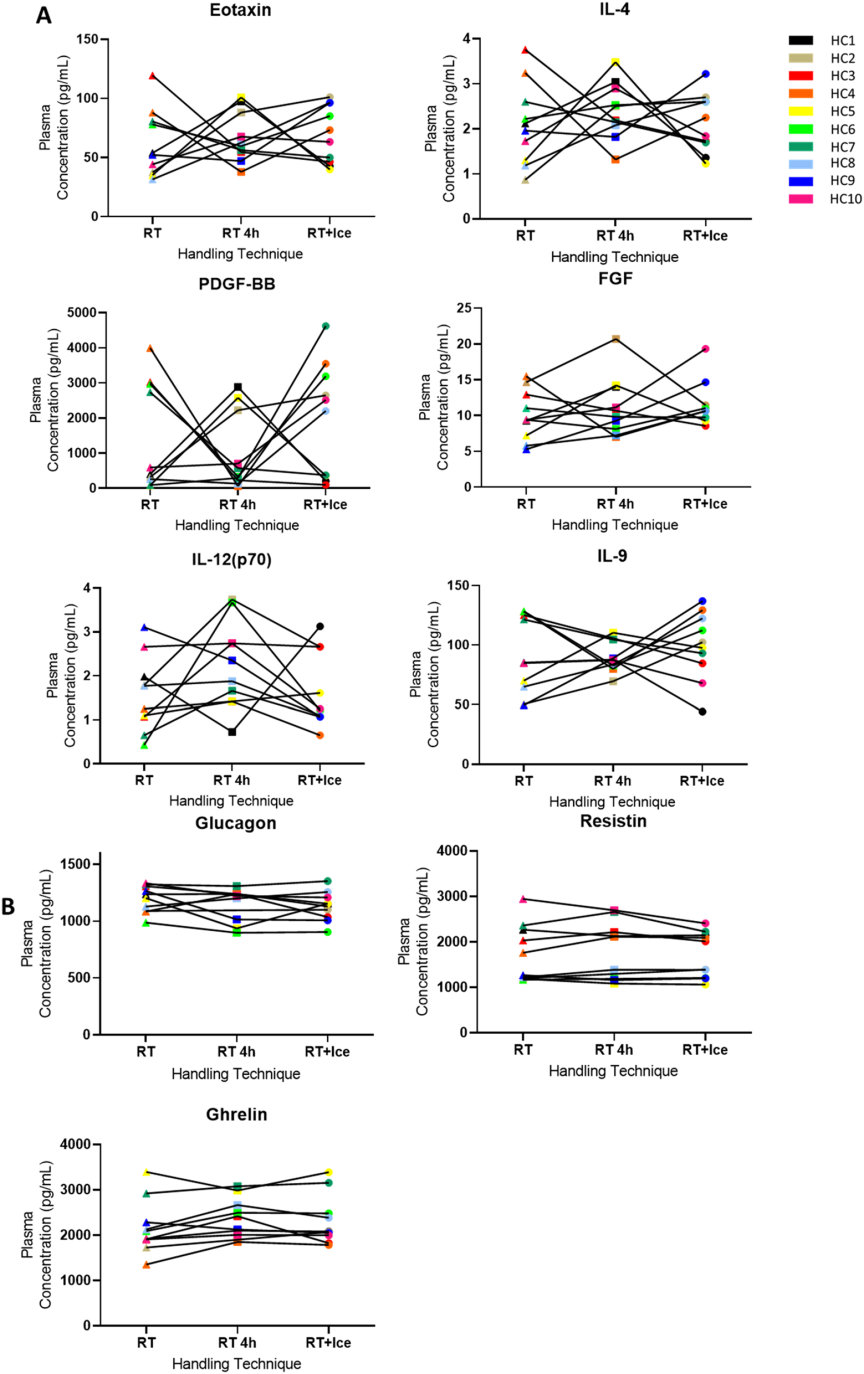
The effects of delay pre-processing protocols on plasma analyte levels. The plasma levels of selected analytes in healthy control samples following three pre-processing protocols are shown. The reference protocol 1 h at room temperature (RT) and two delay protocols, 1 h at RT followed by a further 3 h on ice (RT + Ice) or 1 h at RT followed by a further 3 h at RT (RT 4 h) were compared. (**A**) While no statistically significant differences are observed for some analytes, specific individual samples undergo considerable changes in response to handling variables. (**B**) Some analytes demonstrated minimal changes. Paired t-tests were performed to determine if a significant difference between analyte levels in samples from delay and reference protocols existed.

No significant changes in the mean plasma protein concentrations of the 29 analytes evaluated were observed when plasma was subject to either the RT + Ice protocol or the RT 4 h protocol. Despite the lack of statistically significant changes to the means of each condition, for many of the analytes, the change in the measured analyte concentrations between samples processed in the delayed protocols was apparent for certain individuals (Fig. 4A, Supplementary Figure S4). ROC analysis showed that for 13 analytes (eotaxin, visfatin, FGF-basic, G-CSF, IL-12, IL-17, IL-4, IL-9, IP-10, MCP-1, MIP-1β, PDGF-bb, RANTES), a delay, regardless of the holding temperature, caused misclassification of individuals (AUC values being < 0.7; Table 3, Supplementary Figure S8A). However, GLP-1, glucagon, IL-13, and IL-7 concentrations were less subject to misclassification (AUCs ≥ 0.7) in samples subjected to the RT 4 h protocol versus the RT + Ice protocol (Table 3, Supplementary Figure S8B). Conversely, for IL-1β, PAI-1 and TNFα, measured analyte concentrations resulting from the RT + Ice protocol showed less deviation from the reference protocol compared to the RT 4 h protocol (Table 3, Supplementary Figure S8C). As before, some analytes, namely C-Peptide, ghrelin, GIP, IL-1ra, IL-2, insulin, leptin, MIP-1α, and resistin were largely unaffected by a delay in processing (Fig. 4B, Supplementary Figure S8D), having AUC values of ≥ 0.7 in both delayed protocols (Table 3).

## Discussion

Given the importance of blood as a source of novel biomarkers and the recognition that alterations to sampling, processing or storage can affect biomarker levels, significant efforts have been made to identify optimum protocols for obtaining and storing serum and plasma within clinical studies^10^. SOPs governing the drawing, processing, storage and analysis of blood samples are routinely implemented, with requirements for deviations from SOPs to be recorded. Delays to blood processing are occasionally inevitable, and the paucity of documented evidence on how best to minimise the effects of a delay on blood-borne analytes prompted this study. We included analytes that derive from blood cells, such as certain cytokines, whose levels could alter post blood draw, as they continue to be released from collected cells. We also included analytes that do not derive from blood cells, such as the diabetes-associated proteins, where in vitro release during processing is not expected.

Some analytes, such as leptin and insulin were remarkably stable, demonstrating little change in measured values when either serum or plasma processing was delayed, regardless of whether samples were held on ice or at room temperature during the delay. Notably, for plasma, stable analytes included the cytokines IL-1Ra and IL-2, and diabetes-associated proteins, such as C-Peptide, ghrelin and leptin. Other analytes were much less stable and incurred alterations to their measured serum or plasma values when processing was delayed, regardless of the holding temperature during the delay. Almost half of the analytes measured fell into this category with cytokines being particularly prone to fluctuations following a delay. Thus, while some analytes are highly robust, for many others, a delay, regardless of the holding temperature, introduced variability that could confound biomarker discovery projects.

We observed that the direction of change in analyte levels was occasionally but not always consistent across all subjects. For example, a 3 h delay to plasma processing with samples held on ice led to a systematic increase in the levels of RANTES (Fig. 2A), while for other analytes, such as PDGF-bb, this delay caused increases in some individuals, but decreases in others (Fig. 2A). In addition, the performance of analytes often differed in serum compared to plasma. For example, plasma IL-13 levels were largely unaffected by a processing delay of 3 h at room temperature, while serum IL-13 levels were considerably adversely affected by such a delay. The apparently unpredictable reaction of some analytes to delays makes generalisations difficult. Nonetheless, we recognised that for many studies, samples are collected with the intention of undertaking biomarker discovery, and no such pre-study analyses are possible. We therefore questioned whether holding samples at RT or at 4 °C was generally superior in the event of an unavoidable delay. Bearing in mind that for almost half of all analytes measured in serum or plasma, a delay, regardless of temperature, was detrimental, here we focussed only on analytes where one of the delay protocols yielded AUC ≥ 0.7, while the other yielded AUC < 0.7. In the case of serum samples, six analytes performed better (AUC ≥ 0.7) when held on ice compared to room temperature during a delay (AUC < 0.7) while only one analyte performed better when held at RT compared to ice. This suggests that holding serum samples on ice rather than at RT may be preferable during a delay. For plasma, the outcome was less clear with two analytes performing better when held on ice rather than at RT during a delay, compared with four analytes performing better when held at RT rather than on ice.

It is not recommended that blood be allowed to clot on ice. We observed that doing so made serum difficult to handle and was accompanied by decreased levels of a number of cytokines, supporting the contention that placing serum tubes immediately on ice should be avoided. Placing plasma tubes immediately on ice caused fewer changes. Hennø et al.^6^ also investigated the effects of temperature on the cytokine contents of plasma, making comparisons relative to the plasma cytokine levels of whole blood processed ‘immediately’ after blood draw (T0). They recommended cytokine measurement in plasma that is immediately cooled and rapidly processed, as it consistently resulted in the lowest analyte level that most closely matched their reference protocol (T0). We found, however, that incubation on ice after 1 h at RT significantly increased the plasma concentration of analytes such as MIP-1b, IL-9 and RANTES. Moreover, in serum, we observed that the introduction of ice after 1 h at room temperature caused a significant reduction in the concentration of eotaxin, G-CSF (Fig. 3A), compared to room temperature for 1 h alone (RT). This reduction in concentration suggests that ice can also negatively impact specific analyte levels and the lowest measurable value may not necessarily be the closest to in vivo value.

Our study was not designed to elucidate mechanisms by which holding temperature affects protein concentration. Future studies could take a mechanistic approach by measuring the levels of haemolysis and/or platelet activation within significantly altered samples. Thousands of proteins have been identified within the platelets of healthy humans and can exist with large levels of variation between individuals^11,12^. Furthermore, the use of EDTA for the collection of plasma has been shown to cause strong inter-patient variability on the in vitro activation of platelets. Importantly, EDTA induced activation is elevated by the immediate cooling of whole blood^13^. This combination of diverse platelet protein compositions and varied levels of activation between individuals could account for the large levels of inter-individual variation observed in this study. Measurement of serotonin release, a characteristic marker of platelet activation^14^ alongside the use of flow cytometry to determine CD62P levels would provide a direct measure of α-granulin release^15^. It is most likely multiple mechanisms and cell types collectively influence the response of samples to holding temperatures.

In conclusion, our results indicate that some analytes are highly robust and are unaffected by short delays to processing regardless of the holding temperature, while for others a delay at room temperature or on ice introduced considerable variability. We observed changes that were analyte-specific or subject-specific and in consequence, recommending an optimal SOP for holding samples during a delay to processing is not possible. Rather, the procedure to follow in the event of delays should be clearly outlined in study SOPs and deviations from SOPs should be logged as quality incidents that may require a subsequent subgroup analysis. Practicality and robustness are important attributes of any clinical biomarker. If the biomarker to be analysed is known prior to study development, we advise performing pre-study evaluation to determine the optimum protocols for that biomarker should delays to processing arise. Under circumstances where biomarkers for analyses are unknown, strict adherence to SOPs is vitally important, to enable biomarker signals to be detected above noise.

## Methods

### Blood extraction and processing

Blood collection and analysis were performed following ethics protocols approved by the UK Health Research Authority (London – South East Research Ethics Committee, Ethics Identifier:16/LO/1630) and in accordance with the Declaration of Helsinki. Following written informed consent, 10 healthy volunteers each donated six 10 mL blood samples, including three 4 mL plastic vacutainer tubes (BD Vacutainer Serum Tube, Becton, Dickinson UK Ltd.) for the processing of serum and three 10 mL EDTA-containing plastic tubes (K2 EDTA BD Vacutainer, Becton, Dickinson UK Ltd.) for the processing of plasma. Blood was collected using BD Vacutainer Safety-Lok, needle gauge 23, gently rotated 10 times by hand, and immediately subjected to one of the following conditions: incubation at room temperature for 1 h (RT); incubation at room temperature for 1 h followed by 3 h on crushed ice (RT + Ice) or incubation on crushed ice for 1 h (Ice). For both plasma and serum tubes, samples were centrifuged, at room temperature for 10 min at 1,300×*g*. The resulting serum fraction was immediately removed and aliquoted into cryotubes. The contents of EDTA tubes were aliquoted into 1.5 mL Eppendorf tubes and centrifuged at 16,000 × g for 1 min and the subsequent plasma fraction was collected into cryotubes. Both serum and plasma cryotubes were stored at −80 °C.

All Samples underwent two freeze–thaw cycles. Serum and plasma concentrations of C-peptide, ghrelin, GIP, GLP-1, insulin, leptin, PAI-1, resistin, visfatin, IL-8, IL-9, IFN-g, MIP1-α, MIP1-β, PDGF-BB and RANTES were measured in duplicate by Luminex assay.

In a separate experiment, six 10 mL blood samples were collected as before, each from a different group of ten healthy volunteers, for the processing of both serum and plasma. Blood was immediately subjected to two of the conditions above; incubation at room temperature for 1 h (RT); incubation at room temperature for 1 h followed by 3 h on crushed ice (RT + Ice); and an additional condition comprising incubation at room temperature for 4 h (4RT) was introduced. Samples were processed as before, and stored at −80 °C. All samples underwent two freeze–thaw cycles. Serum and plasma concentrations of C-peptide, ghrelin, GIP, GLP-1, insulin, leptin, PAI-1, glucagon, resistin, visfatin, eotaxin, FGF, G-CSF, IL-12(p70), IL-13, 1L-17, IL-1β, IL-1ra, IL-2, IL-4, IL-6, IL-7, IL-9, IP-10, MCP-1(MCAF), MIP1-α, MIP1-β, PDGF-BB, RANTES and TNF-α were measured in duplicate by Luminex assay.

### Luminex assay

For analyte measurement, Luminex multi-plex assays were performed, as described previously^16^ using the Bio-Plex pro diabetes 10-plex, a human cytokine custom-made 11-plex and human cytokine 27-plex plates (Bio-Rad Laboratories Inc. Bio-Rad Laboratories Ltd, Hercules, CA, USA)^16^. Briefly, serially diluted standards and test samples diluted 1 in 4 with sample diluent and 50 µL added to a Bio-Plex Pro flat-bottomed plate containing antibody-coupled beads. Plates were shaken (900 rpm) at room temperature for 1 min followed by 300 rpm for 30 min. Following washing using a Bio-Plex Pro wash station, secondary antibodies (25 µL) were added and the plates incubated as before, washed again and Streptavidin-PE (50 µL) added. Plates were shaken at 900 rpm for 1 min at room temperature followed by 300 rpm for 15 min. Assay buffer (125 µL) was added to each well before reading plates on a Bio-Plex 200 instrument. Standard curves, fit using a five parameter logistic regression, were used to quantify proteins in test samples. Each sample was assayed in duplicate.

### Statistical analysis

Interplate reproducibility was determined using three quality controls with a coefficient of variance (CV) limit of 20% applied. A CV limit of 20% was applied to duplicate samples. SPSS (REF) was used to perform paired t-tests with Holm’s post-hoc multiple comparison correction. A significance limit of *p* ≤ 0.05 was used. Data were visualised using GraphPad Prism 6 (GraphPad Software, La Jolla California USA). R version 3.6.2^17^ was used to generate ROC curves and obtain AUC values, packages used were tidyverse, plotROC and flextable.

## Supplementary information

Supplementary information.
