# Supplementary material for: The holding temperature of blood during a delay to processing can affect serum and plasma protein measurements
Source: Sci Rep. 2021 Mar 22;11:6487. doi: 10.1038/s41598-021-85052-5 (PMC7985364; doi:10.1038/s41598-021-85052-5)
Supplement: Supplementary file 1 — Supplementary information. [file 41598_2021_85052_MOESM1_ESM.docx]

The holding temperature of blood during a delay to processing can affect serum and plasma protein measurements.

Milton Ashworth^1∞^, Benjamin Small^1∞^, Lucy Oldfield^1^, Anthony Evans^1^, William Greenhalf^1^, Christopher Halloran^1^, Eithne Costello^1^*.

^1^Department of Molecular and Clinical Cancer Medicine, University of Liverpool, UK

^∞^ Contributed equally to this manuscript.


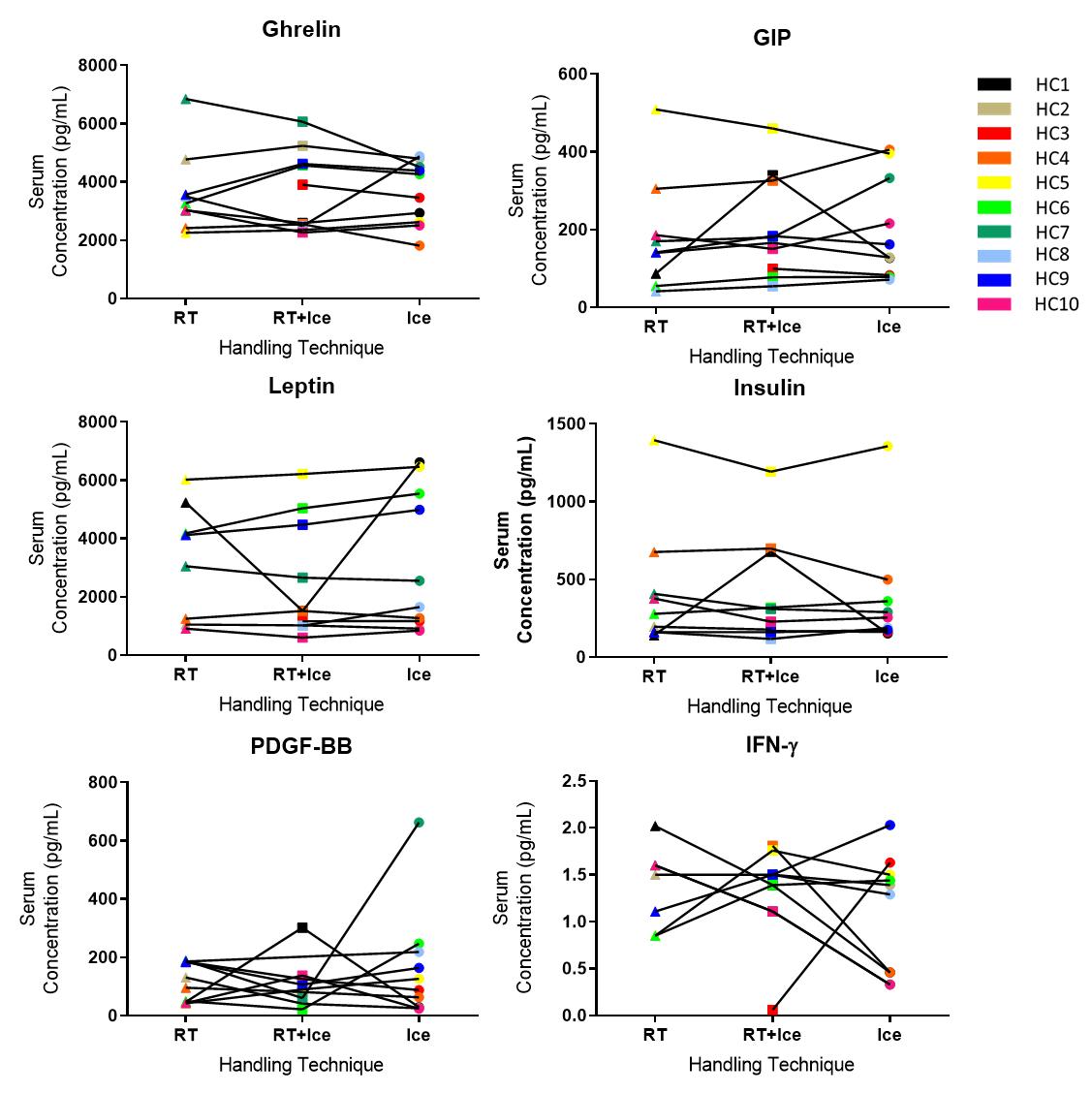


**Supplementary Figure S1. The effects of alternative pre-processing protocols on serum analyte levels.**

The serum levels of selected analytes in healthy control samples following three pre-processing protocols are shown. The reference protocol, 1 h at room temperature (RT) and two alternative protocols, 1 h at room temperature followed by a further three hrs on ice (RT + Ice) or 1 h on ice (Ice) were compared.


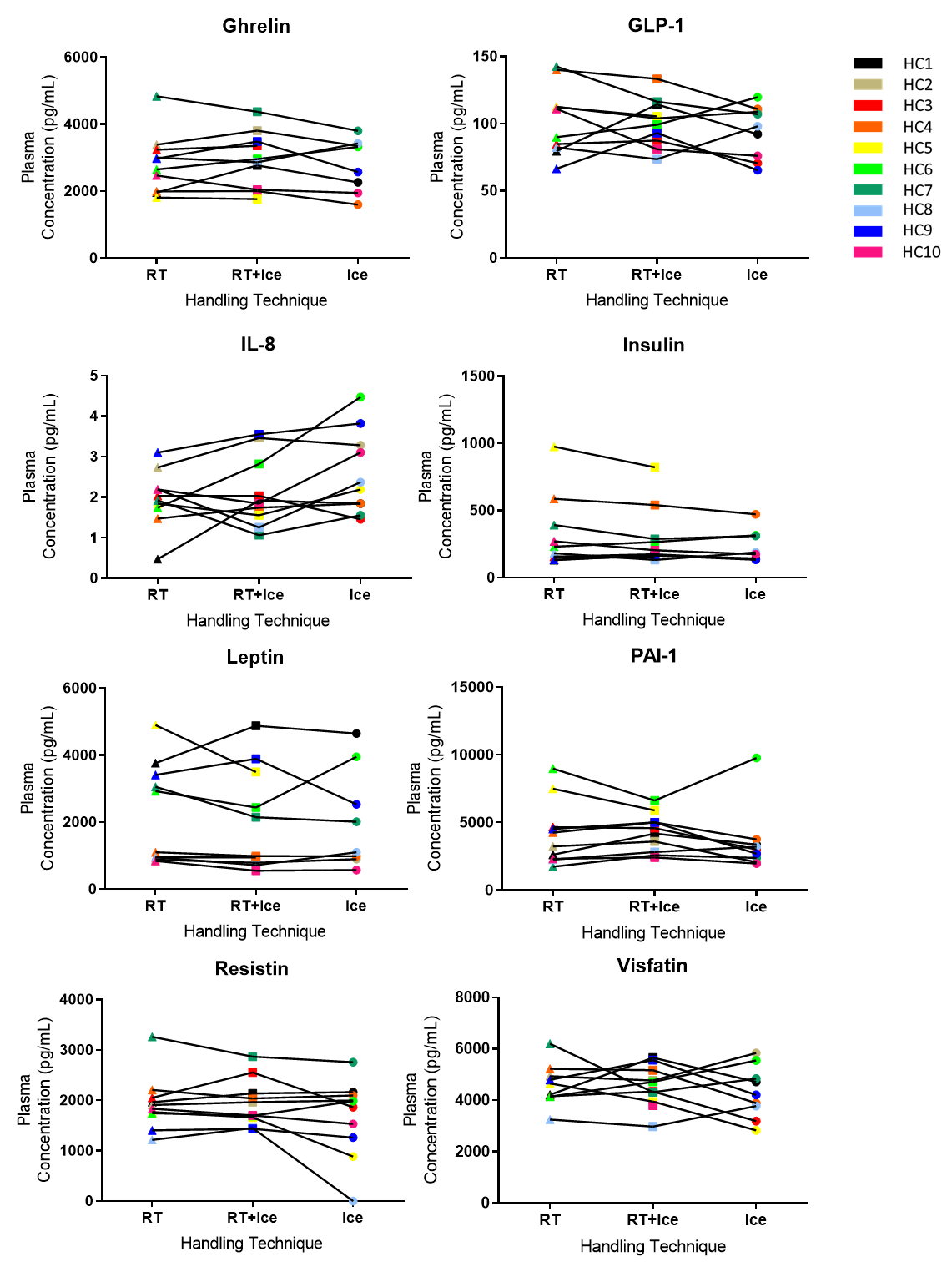


**Supplementary Figure S2. The effects of alternative pre-processing protocols on plasma analyte levels.**

The plasma levels of selected analytes in healthy control samples following three pre-processing protocols are shown. The reference protocol, 1 h at room temperature (RT) and two alternative protocols, 1 h at room temperature followed by a further three hrs on ice (RT + Ice) or 1 h on ice (Ice) were compared.


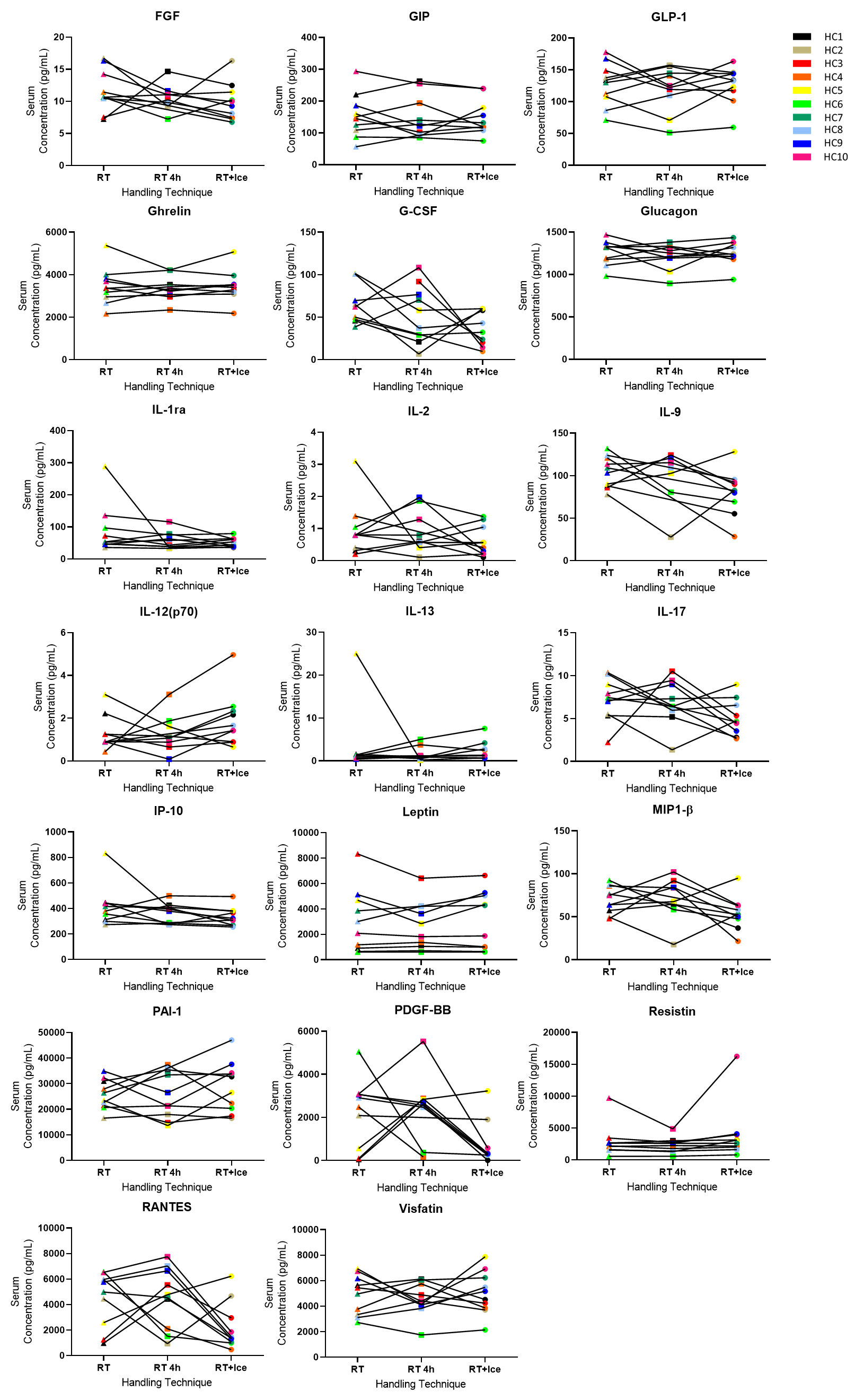


**Supplementary Figure S3. The effects of a delay and pre-processing protocols on serum analyte levels.**

The serum levels of selected analytes in healthy control samples following three pre-processing protocols are shown. The reference protocol, 1 h at room temperature (RT) and two delay protocols, 1 h at room temperature followed by a further three hrs on ice (RT + Ice) or 1 h at room temperature followed by a further 3 hrs at room temperature (RT 4h) were compared.


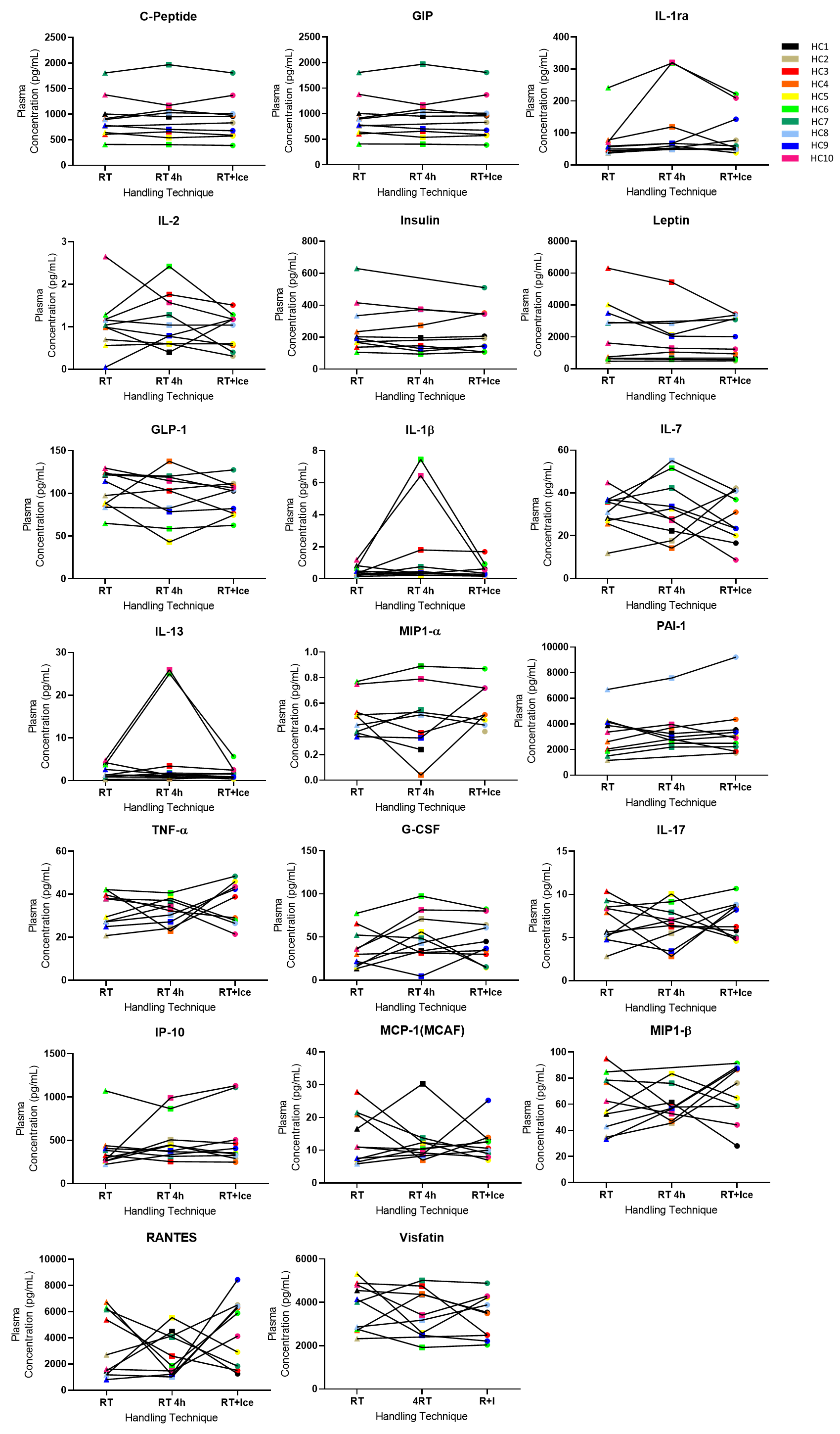


**Supplementary Figure S4. The effects of delay pre-processing protocols on plasma analyte levels.** The plasma levels of selected analytes in healthy control samples following three pre-processing protocols are shown. The reference protocol, 1 h at room temperature (RT) and two delay protocols, 1 h at room temperature followed by a further three hrs on ice (RT + Ice) or 1 h at room temperature followed by a further 3 hrs at room temperature (RT 4h) were compared.


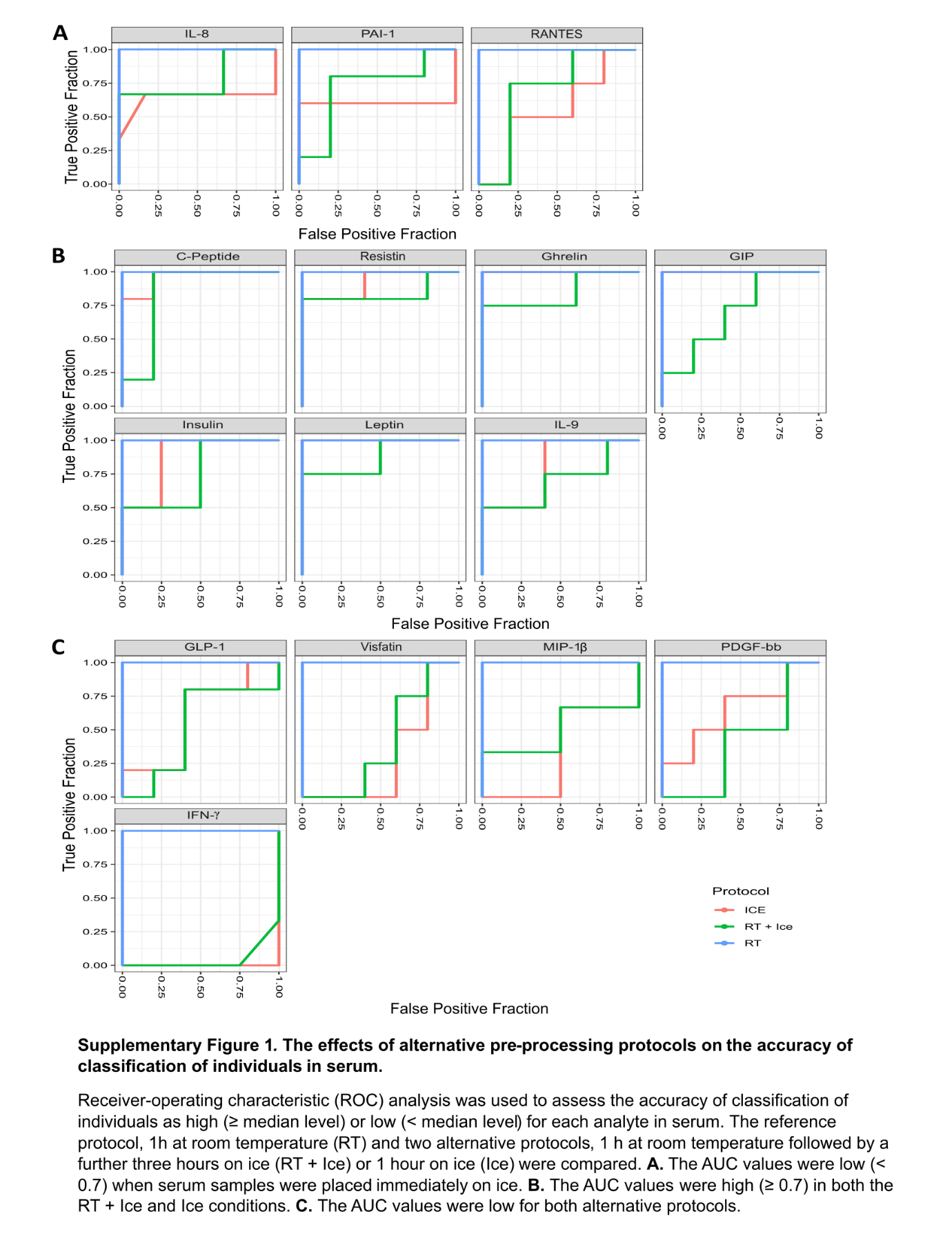


**Supplementary Figure S5. The effects of alternative pre-processing protocols on the accuracy of classification of individuals in serum.**

Receiver-operating characteristic (ROC) analysis was used to assess the accuracy of classification of individuals as high (≥ median level) or low (< median level) for each analyte in serum. The reference protocol, 1 h at room temperature (RT) and two alternative protocols, 1 h at room temperature followed by a further three hrs on ice (RT + Ice) or 1 h on ice (Ice) were compared. **A.** The AUC values were low (< 0.7) only when serum samples were placed immediately on ice. **B.** The AUC values were high (≥ 0.7) in both the RT + Ice and Ice protocols. **C.** The AUC values were low for both alternative protocols.


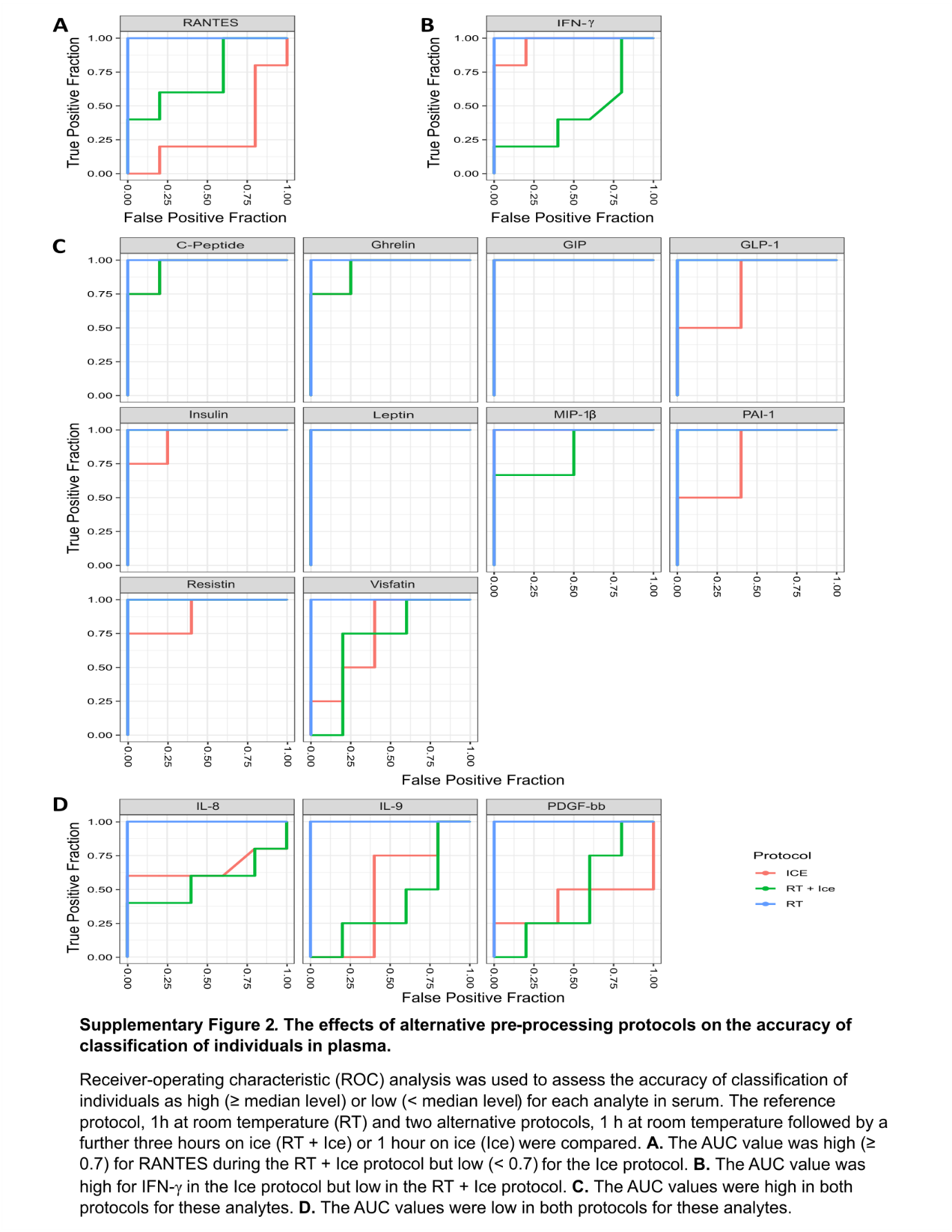


**Supplementary Figure S6. The effects of alternative pre-processing protocols on the accuracy of classification of individuals in plasma.**

Receiver-operating characteristic (ROC) analysis was used to assess the accuracy of classification of individuals as high (≥ median level) or low (< median level) for each analyte in plasma. The reference protocol, 1 h at room temperature (RT) and two alternative protocols, 1 h at room temperature followed by a further three hrs on ice (RT + Ice) or 1 h on ice (Ice) were compared. **A.** The AUC value was high (≥ 0.7) for RANTES in the RT + Ice protocol but low (< 0.7) in the Ice protocol. **B.** The AUC value was high for IFN-γ in the Ice protocol but low in the RT + Ice protocol. **C.** The AUC values were high for both alternative protocols for these analytes. **D.** The AUC values were low in both protocols for these analytes.


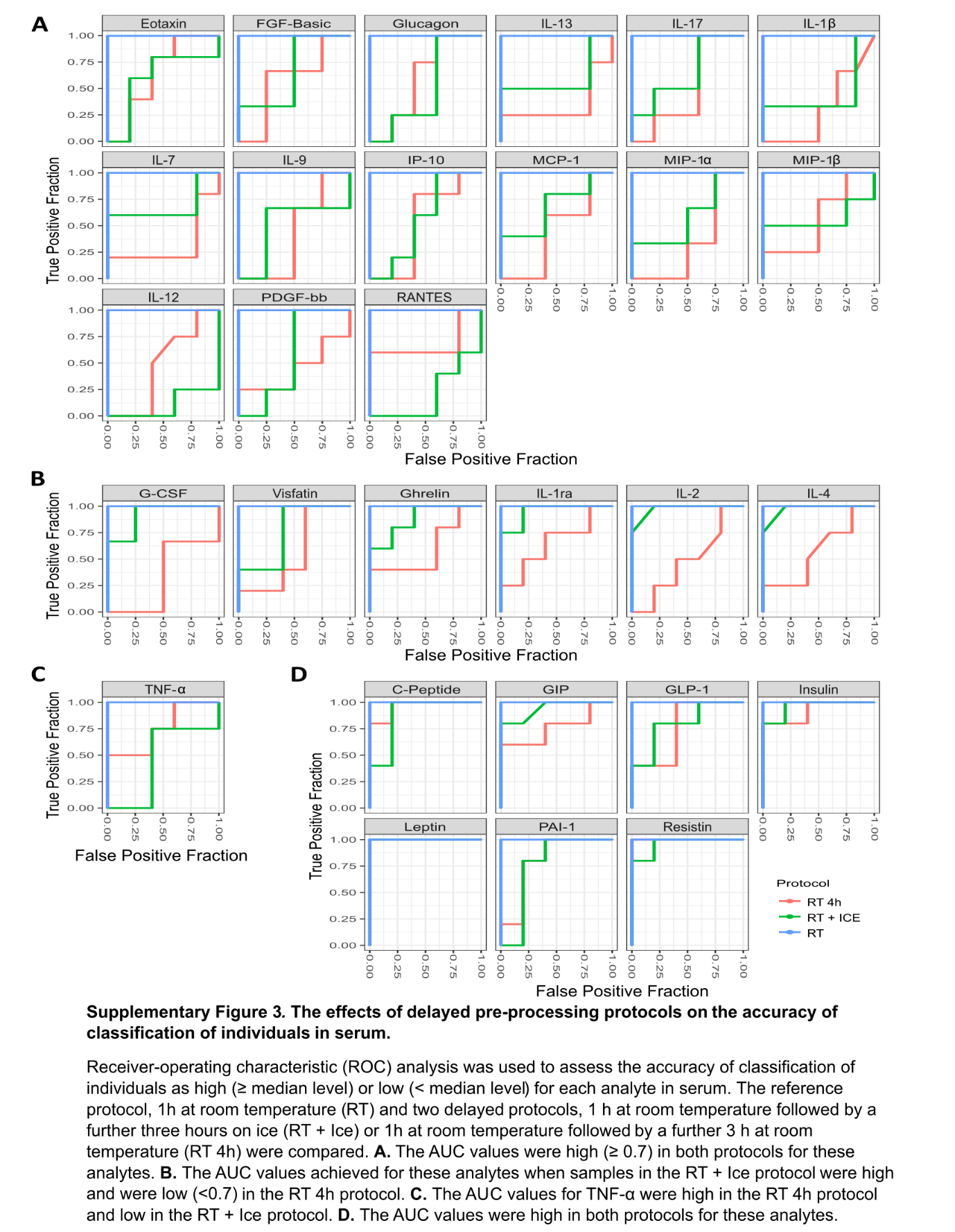


**Supplementary Figure S7. The effects of delayed pre-processing protocols on the accuracy of classification of individuals in serum.**

Receiver-operating characteristic (ROC) analysis was used to assess the accuracy of classification of individuals as high (≥ median level) or low (< median level) for each analyte in serum. The reference protocol, 1h at room temperature (RT) and two delayed protocols, 1 h at room temperature followed by a further three hrs on ice (RT + Ice) or 1 h at room temperature followed by a further three hrs at room temperature (RT 4h) were compared. **A.** The AUC values were high (≥ 0.7) in both protocols for these analytes. **B.** The AUC values for these analytes in the RT + Ice protocol were high but were low (< 0.7) in the RT 4h protocol. **C.** The AUC values for TNF-a were high in the RT 4h protocol and low in the RT + Ice protocol. **D.** The AUC values were high in both protocols for these analytes.


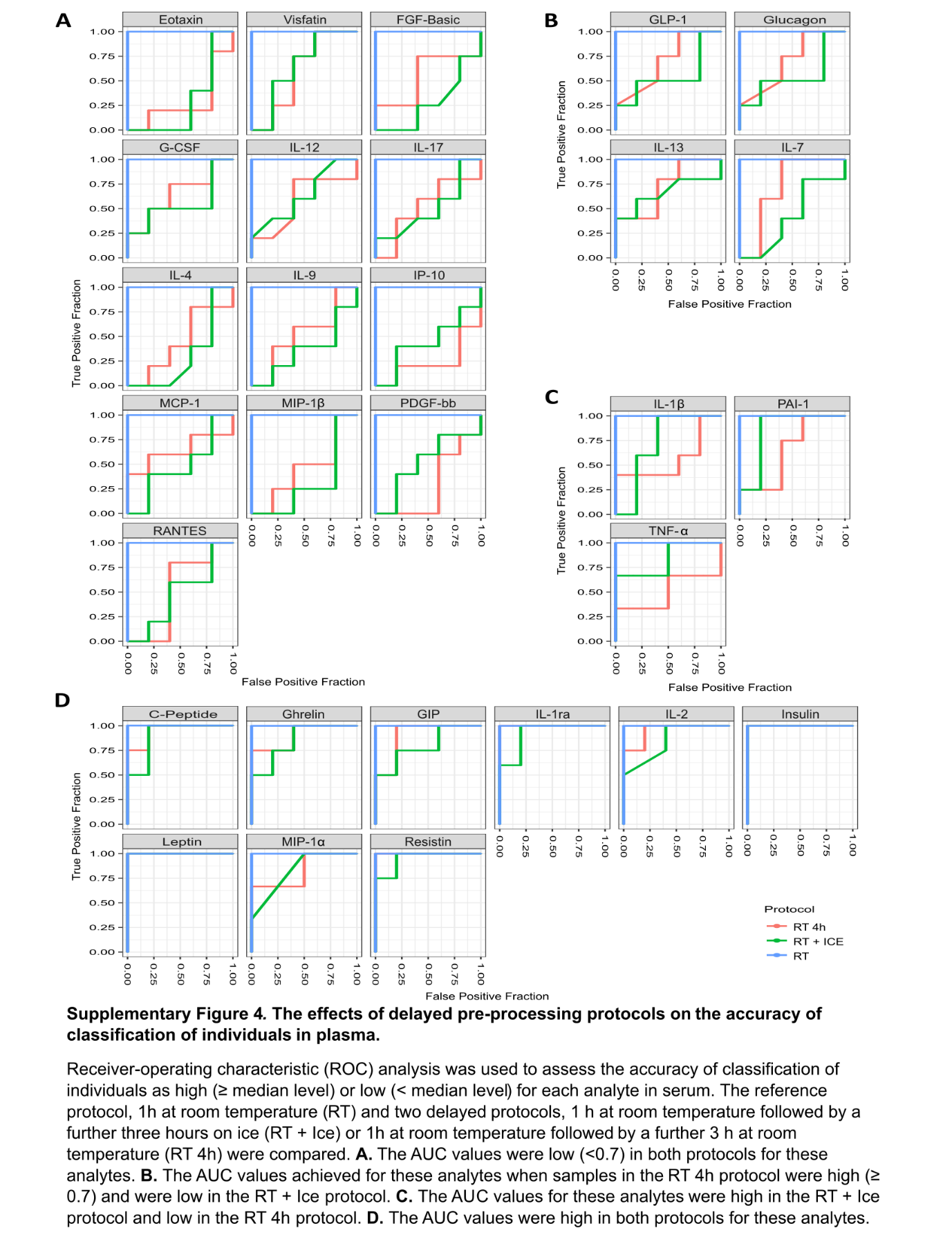


**Supplementary Table S1. The mean analyte concentration with standard error of the mean (SEM) from the first experiment.**

**Supplementary Figure S8. The effects of delayed pre-processing protocols on the accuracy of classification of individuals in plasma.**

Receiver-operating characteristic (ROC) analysis was used to assess the accuracy of classification of individuals as high (≥ median level) or low (< median level) for each analyte in plasma. The reference protocol, 1h at room temperature (RT) and two delayed protocols, 1 h at room temperature followed by a further three hrs on ice (RT + Ice) or 1 h at room temperature followed by a further three hrs at room temperature (RT 4h) were compared. **A.** The AUC values were low (< 0.7) in both protocols for these analytes. **B.** The AUC values for these analytes in the RT 4h protocol were high (≥ 0.7) but were low in the RT + Ice protocol. **C.** The AUC values for these analytes were high in the RT + Ice protocol and low in the RT 4h protocol. **D.** The AUC values were high in both protocols for these analytes.

|  | Plasma [pg/mL] | | | | | | Serum [pg/mL] | | | | | |
| --- | --- | --- | --- | --- | --- | --- | --- | --- | --- | --- | --- | --- |
|  | RT | | RT + Ice | | Ice | | RT | | RT + Ice | | Ice | |
| Cytokine | Mean | SEM | Mean | SEM | Mean | SEM | Mean | SEM | Mean | SEM | Mean | SEM |
| C-Peptide | 806.65 | 115.08 | 788.83 | 105.04 | 779.08 | 101.19 | 1007.51 | 273.02 | 1082.37 | 231.52 | 1020.94 | 251.22 |
| Ghrelin | 2907.63 | 326.17 | 3036.87 | 291.18 | 2785.32 | 283.12 | 3636.15 | 469.16 | 3642.04 | 491.66 | 3641.52 | 385.04 |
| GIP | 307.46 | 72.68 | 280.42 | 62.23 | 275.35 | 66.81 | 182.00 | 48.68 | 215.57 | 44.29 | 213.32 | 44.07 |
| GLP-1 | 3839.40 | 734.98 | 4089.74 | 460.34 | 3579.35 | 798.73 | 119.34 | 9.32 | 121.36 | 8.25 | 116.72 | 5.91 |
| IFN-g | 1.67 | 0.13 | 1.63 | 0.14 | 1.73 | 0.14 | 1.36 | 0.17 | 1.39 | 0.09 | 1.07 | 0.26 |
| IL-8 | 2.12 | 0.28 | 1.97 | 0.22 | 2.59 | 0.32 | 2.60 | 0.20 | 2.44 | 0.28 | 1.72 | 0.25 |
| IL-9 | 32.37 | 1.29 | 23.47 | 2.53 | 27.35 | 2.20 | 44.27 | 1.74 | 47.15 | 1.34 | 31.16 | 1.66 |
| Insulin | 260.38 | 55.79 | 242.09 | 46.51 | 234.10 | 42.76 | 450.72 | 148.89 | 463.99 | 129.74 | 410.13 | 141.04 |
| Leptin | 2118.35 | 452.03 | 2050.10 | 571.66 | 2087.94 | 536.77 | 3231.83 | 701.30 | 2885.78 | 739.60 | 3651.85 | 888.93 |
| MIP-1β | 54.56 | 3.35 | 38.78 | 6.26 | 47.63 | 5.14 | 80.24 | 5.19 | 79.97 | 4.19 | 57.02 | 2.87 |
| PAI-1 | 3839.40 | 734.98 | 4089.74 | 460.34 | 3579.35 | 798.73 | 24332.64 | 2753.48 | 26093.86 | 2669.41 | 8725.12 | 1195.06 |
| PDGF-bb | 1324.24 | 184.45 | 947.20 | 227.55 | 710.07 | 224.07 | 108.06 | 21.91 | 107.94 | 27.41 | 159.37 | 67.69 |
| RANTES | 2884.75 | 353.93 | 1003.09 | 132.26 | 2066.99 | 333.38 | 7216.38 | 410.43 | 7658.93 | 200.78 | 2990.11 | 299.97 |
| Resistin | 2016.07 | 172.58 | 2006.22 | 153.75 | 1838.54 | 182.74 | 2311.44 | 358.67 | 2193.69 | 350.67 | 1950.64 | 157.51 |
| Visfatin | 4619.44 | 275.61 | 4607.35 | 279.16 | 4315.55 | 338.31 | 5401.40 | 394.03 | 5627.37 | 447.76 | 5251.80 | 346.66 |

*(SEM = Standard Error of the Mean)*

**Supplementary Table S2. The mean analyte concentration with standard error of the mean (SEM) from the second experiment.**

|  | Plasma [pg/mL] | | | | | | Serum [pg/mL] | | | | | |
| --- | --- | --- | --- | --- | --- | --- | --- | --- | --- | --- | --- | --- |
|  | RT | | RT + Ice | | 4h RT | | RT | | RT + Ice | | 4h RT | |
| Cytokine | Mean | SEM | Mean | SEM | Mean | SEM | Mean | SEM | Mean | SEM | Mean | SEM |
| C-Peptide | 938.27 | 142.34 | 928.16 | 147.97 | 946.18 | 154.90 | 966.26 | 141.90 | 940.21 | 129.47 | 955.43 | 142.55 |
| Eotaxin | 61.99 | 9.01 | 69.41 | 7.65 | 67.14 | 6.75 | 75.40 | 7.84 | 50.16 | 2.38 | 73.93 | 8.87 |
| FGF-Basic | 10.14 | 1.23 | 11.70 | 1.11 | 10.91 | 1.43 | 11.87 | 1.49 | 10.54 | 1.14 | 10.55 | 0.87 |
| GCSF | 37.43 | 7.71 | 47.61 | 8.58 | 51.93 | 9.43 | 65.89 | 9.74 | 41.71 | 7.06 | 47.30 | 13.02 |
| Ghrelin | 2212.58 | 201.82 | 2350.31 | 190.99 | 2414.14 | 144.32 | 3456.95 | 275.18 | 3474.01 | 229.49 | 3363.34 | 176.84 |
| GIP | 294.19 | 52.69 | 266.54 | 50.21 | 257.55 | 49.61 | 153.30 | 21.57 | 148.05 | 17.52 | 147.00 | 21.14 |
| GLP-1 | 1208.05 | 39.67 | 1134.16 | 45.26 | 1144.24 | 50.53 | 127.18 | 10.67 | 126.44 | 9.20 | 119.65 | 11.06 |
| Glucagon | 1208.05 | 39.67 | 1134.16 | 45.26 | 1144.24 | 50.53 | 1269.68 | 49.78 | 1250.93 | 48.42 | 1210.05 | 51.76 |
| IL-12 | 1.58 | 0.27 | 1.64 | 0.27 | 2.24 | 0.31 | 1.31 | 0.28 | 1.92 | 0.44 | 1.28 | 0.29 |
| IL-13 | 2.14 | 0.51 | 1.76 | 0.49 | 6.25 | 3.22 | 3.74 | 2.67 | 2.40 | 0.76 | 1.60 | 0.55 |
| IL-17 | 6.81 | 0.76 | 7.16 | 0.66 | 6.55 | 0.72 | 6.86 | 0.77 | 5.39 | 0.65 | 6.84 | 0.91 |
| IL-1b | 0.49 | 0.10 | 0.53 | 0.15 | 1.85 | 0.87 | 1.19 | 0.93 | 0.72 | 0.24 | 0.34 | 0.11 |
| IL-1ra | 71.67 | 19.38 | 94.96 | 22.16 | 115.63 | 34.65 | 91.22 | 26.85 | 50.25 | 4.90 | 57.78 | 9.29 |
| IL-2 | 1.07 | 0.24 | 0.96 | 0.14 | 1.16 | 0.22 | 0.91 | 0.29 | 0.62 | 0.16 | 0.90 | 0.22 |
| IL- 4 | 2.10 | 0.29 | 2.12 | 0.21 | 2.40 | 0.20 | 2.40 | 0.23 | 1.79 | 0.14 | 2.15 | 0.29 |
| IL-7 | 31.42 | 2.85 | 28.40 | 3.71 | 32.44 | 4.33 | 32.94 | 5.20 | 27.93 | 4.15 | 31.03 | 4.40 |
| IL-9 | 90.79 | 10.23 | 99.07 | 9.01 | 90.02 | 4.08 | 103.86 | 7.65 | 91.17 | 7.02 | 97.27 | 12.81 |
| Insulin | 223.67 | 36.55 | 218.54 | 38.94 | 212.21 | 40.49 | 357.97 | 64.26 | 341.86 | 58.11 | 328.98 | 61.08 |
| IP-10 | 395.41 | 78.33 | 518.46 | 103.31 | 490.92 | 76.89 | 420.04 | 50.02 | 335.05 | 22.88 | 363.69 | 24.69 |
| Leptin | 2548.16 | 716.63 | 1919.76 | 438.27 | 2005.98 | 562.90 | 3044.62 | 791.11 | 3074.27 | 718.64 | 2688.81 | 605.02 |
| MCP-1 | 13.58 | 2.43 | 12.19 | 1.62 | 12.27 | 2.11 | 15.75 | 2.31 | 10.18 | 0.98 | 14.41 | 1.99 |
| MIP-1α | 0.55 | 0.06 | 0.60 | 0.06 | 0.49 | 0.11 | 0.68 | 0.11 | 0.43 | 0.09 | 0.75 | 0.15 |
| MIP-1β | 59.01 | 7.06 | 65.92 | 7.00 | 59.75 | 4.19 | 66.93 | 5.73 | 53.71 | 7.62 | 71.23 | 9.25 |
| PAI-1 | 3375.29 | 534.45 | 3666.17 | 736.65 | 3518.31 | 541.32 | 25740.20 | 1831.82 | 28820.97 | 3125.63 | 25785.85 | 2911.24 |
| PDGF-bb | 1447.29 | 485.26 | 1976.52 | 511.86 | 1000.31 | 349.35 | 2238.03 | 636.24 | 657.91 | 371.92 | 2742.93 | 491.25 |
| RANTES | 3353.56 | 780.35 | 4517.39 | 800.15 | 2750.96 | 524.47 | 4506.82 | 676.38 | 2248.03 | 583.50 | 4531.81 | 744.90 |
| Resistin | 1801.78 | 213.95 | 1747.68 | 175.21 | 1848.31 | 215.77 | 2924.02 | 794.01 | 3982.42 | 1399.58 | 2324.26 | 373.16 |
| TNF-α | 33.62 | 2.69 | 36.00 | 3.28 | 31.87 | 2.08 | 35.64 | 3.06 | 27.90 | 2.28 | 35.28 | 3.62 |
| Visfatin | 4006.23 | 332.75 | 3448.75 | 333.03 | 3563.94 | 370.11 | 4886.52 | 490.29 | 5016.19 | 533.88 | 4545.50 | 409.85 |

*(SEM = Standard Error of the Mean)*
